# Reduction of the planning target volume with daily online adaptive radiotherapy in bladder cancer

**DOI:** 10.1007/s00066-025-02397-w

**Published:** 2025-04-15

**Authors:** Levente Varga, Ádám Gáldi, Domonkos Szegedi, András Herein, Dóra Pulugor, István Nahaji, László Gesztesi, Kliton Jorgo, Zoltán Takácsi Nagy, Csaba Polgár, Zsuzsa Kocsis, Tibor Major, Péter Ágoston

**Affiliations:** 1 Center of Radiotherapy, National Institute of Oncology, Budapest, Hungary; 2 Faculty of Medicine, Semmelweis University, Budapest, Hungary; 3 Department of Oncology, Semmelweis University, Budapest, Hungary; 4 National Tumor Biology Laboratory, National Institute of Oncology, Budapest, Hungary; 5 Department of Radiobiology and Diagnostic Onco-Cytogenetics, Centre of Radiotherapy, National Institute of Oncology, Budapest, Hungary; 6 Doctoral College, Semmelweis University, Budapest, Hungary; 7 Ráth György Street 7–9, Budapest, Hungary

**Keywords:** Re-optimization, Cone Beam CT, Bladder cancer, Artificial intelligence, External beam

## Abstract

**Introduction:**

External radiation therapy for bladder cancer requires large planning target volumes (PTVs) due to the daily anatomy of the bladder. Online adaptive radiotherapy (oART) can reduce the PTV by considering daily anatomical changes.

**Patients and methods:**

We performed oART in 8 patients with muscle-invasive bladder cancer between June 10, 2022, and April 14, 2023, on an Ethos linear accelerator (Varian, Palo Alto, USA). Using the 496 cone-beam computed tomography (CBCT) images of the fractions, we retrospectively compared the differences in volumetric changes between oART and image-guided and intensity-modulated radiotherapy (IGRT/IMRT). According to our local protocol, for oART, a patient-specific PTV margin was created based on the intrafractional clinical target volume (CTV) changes observed during the first three fractions.

**Results:**

The average duration of treatment was 14.8 min (range 7–49 min). The average volume of the PTV with oART and IGRT/IMRT was 296.8 cm^3^ (range 114.5–810.4 cm^3^) and 416.5 cm^3^ (range 188.2–991.3 cm^3^), respectively, representing a 30% reduction with oART. This new technique resulted in an average reduction of 43.9% in the volume of unnecessarily irradiated healthy tissues. Geometrical miss of the CTV occurred in 13 fractions with IGRT/IMRT, with an average of 9.4 cm^3^ of missed volume (range 0.4–56.4 cm^3^, standard deviation [SD] 15.73), for oART in 7 fractions, with an average missed volume of 4 cm^3^ (range 0.4–21.2 cm^3^, SD: 7.6).

**Conclusion:**

The use of patient-specific margins in oART allows for reduction of the PTV and dose to healthy tissues while achieving equal or better target coverage.

## Introduction

In the oncological treatment of bladder cancer, radiation therapy (RT) plays a significant role. In the curative treatment of invasive bladder cancer, radical cystectomy is considered the gold standard, but the multidisciplinary approach to preserve the bladder (trimodal therapy) is gaining increasing recognition [1]. Initially, RT was included as part of trimodal therapy, but recent studies have explored its combination with immunotherapy, examining its immunomodulatory effects [2, 3]. In palliative treatments, radiation therapy has always played a crucial role.

Due to the increasing emphasis on RT, achieving appropriate clinical target volume (CTV) coverage while minimizing side effects has become more important. The intensive technological developments in recent decades, such as computer tomography (CT) or magnetic resonance (MR) image-guided radiation therapy (IGRT) and intensity-modulated radiation therapy (IMRT), have greatly contributed to the success of RT. Nevertheless, the movement and gas content of the bowels and the fullness of the rectum and the bladder can significantly alter the daily anatomical positions of the organs during bladder RT, which poses challenges for radiation oncologists. In the radiation treatment of bladder cancer, movements of the bladder wall of up to 3.5 cm can occur in some cases [4, 5]. Therefore, despite IGRT, a substantial safety margin is still required to ensure proper dose coverage, which results in unnecessary dose exposure to the organs at risk in the surrounding tissue. Adaption to the daily variations in anatomical conditions would enable a significant reduction in the dose exposure of healthy tissues while allowing for an increase in the therapeutic dose delivered to the target area.

Consequently, various adaptive radiation therapy (ART) approaches have been developed, particularly in the context of bladder cancer treatment. The common feature of these techniques, in contrast to conventional IGRT, is their ability not only to detect anatomical changes but also to adapt to them to varying extents. The offline adaptive option, the composite target volume, has paramount importance. In this approach, cone-beam computed tomography (CBCT) images acquired during the first week of treatment are used to create a new adaptive clinical target volume (CTV). This updated target volume is designed to more accurately reflect the patient’s organ size, shape, and position throughout the treatment period, thereby ensuring enhanced precision in radiotherapy delivery [6]. Among the online adaptive techniques, there is the plan-of-the-day (PoD) technique, where multiple plans are prepared for different bladder filling conditions, and the most suitable one is selected day by day. The other option is planning based on daily anatomy, i.e., daily online re-optimization adaptive bladder treatment (ReOpt) [7]. With online adaptive radiation therapy (oART), a new plan is created for each patient for each treatment session based on the daily anatomy, thus eliminating interfractional variations. Adaptive radiation therapy has significantly enhanced dose conformity in bladder radiotherapy—a level of precision that has been challenging to achieve with conventional radiotherapy. Studies have consistently demonstrated that all ART techniques surpass conventional RT in terms of therapeutic efficacy, achieving a more favorable therapeutic ratio [8].

Another option is the use of the MR-linac, which integrates MRI with a linear accelerator (linac). This advanced technology allows for real-time imaging and precise adaptation of the treatment plan during each session, thus enabling superior visualization of soft tissues and improved targeting of the tumor while sparing surrounding healthy structures. This approach offers several potential advantages for bladder cancer patients. However, before it can be adopted for routine clinical use, a robust evidence base must be established to determine whether MR-guided RT translates into improved patient outcomes [9].

The ability of oART to adjust treatment plans daily based on real-time imaging allows for more precise targeting of tumors while sparing normal tissues. This is particularly beneficial for bladder cancer patients, who may experience significant variations in tumor position due to bladder filling and other factors.

This paper summarizes our technical experiences with the application of oART in bladder cancer during introduction of the technique in Hungary.

## Patients and methods

In this study, we analyzed data obtained from the curative online adaptive radiation therapy of 8 patients treated between June 10, 2022, and April 14, 2023. The treatments were performed using the Ethos Therapy system (Varian Medical System, Palo Alto, USA), with cone-beam CT (CBCT)-based planning supported by artificial intelligence (AI).

The basic characteristics of the participants are summarized in Table 1.

**Table 1:** Baseline characteristics of participants

Characteristic	*n* = 8
*Age (years)*	68 (52–78)
*Sex*	
Male	7
Female	1
*T stage*	
2	6
3	1
4	1
*Lymph node metastasis*	
Yes	2
No	6
*Histological grade*	
II	2
III	6
*Concurrent chemotherapy*	
Yes	6
No	2

According to our institutional treatment protocol, 3 patients received a dose of 63 Gy in 1.8-Gy fractions five times a week with conventionally fractionated radiation therapy. Another 3 patients, following tumor bed marking with lipiodol, received 63 Gy/2.1 Gy to the tumor bed and 57 Gy/1.9 Gy to the entire bladder using simultaneous integrated boost technique. Each of these patients also underwent at least three cycles of weekly concomitant 40 mg/m^2^ CDDP chemotherapy (3–5 cycles) during the radiation therapy course. One patient, considering his age, underwent hypofractionated radiation therapy without chemotherapy, receiving a dose of 55 Gy in 20 fractions, according to a moderately hypofractionated bladder irradiation protocol [10]. Another patient had previously undergone pelvic radiation therapy; therefore, he received 50 Gy in 2-Gy fractions to the bladder. For patients under the age of 70 (six cases), elective pelvic lymph node region irradiation was also performed.

### Protocol for adaptive radiation therapy

The adaptation process begins with the initial adaptation CBCT scan. After that, quality assessment of the images is performed. In cases where factors such as gas artifacts, a filled bladder, or rectal distension are present and may complicate the planning process, appropriate interventions can be undertaken. Afterward, the contours of the influencer structures (organs that are near the target volume and whose shape and localization can influence the contours of the target volumes) are automatically generated by the Ethos AI system. During adaptive bladder radiotherapy, these influencer structures are none other than the rectum and the bladder itself. Checking the contours of the influencer structures is very important, as the planning system uses them to create a structure-guided deformable image registration (DIR) between the planning CT scans and the adaptation CBCT scans. Based on the original planning CT contours, the gross tumor volume (GTV) and CTV are propagated from the planning CT to the CBCT using DIR. These are then checked by radiotherapy technologists (RTT) and radiation oncologists together. After this, using elastic DIR, the system creates the contours of the organs at risk (OARs). Once the contours have been created and checked, the system generates a synthetic CT by deforming the planning CT based on the daily CBCT into the current geometry. Cone-beam CT images cannot be used directly for dose calculation due to their lower contrast-to-noise ratios, issues with the conversion from Hounsfield units (HUs) to tissue density, and increased motion artifacts [11]. Therefore, this synthetic CT applies the HUs of the planning CT to provide the density information for dose calculations performed in the real treatment geometry.

**Fig. 1:**
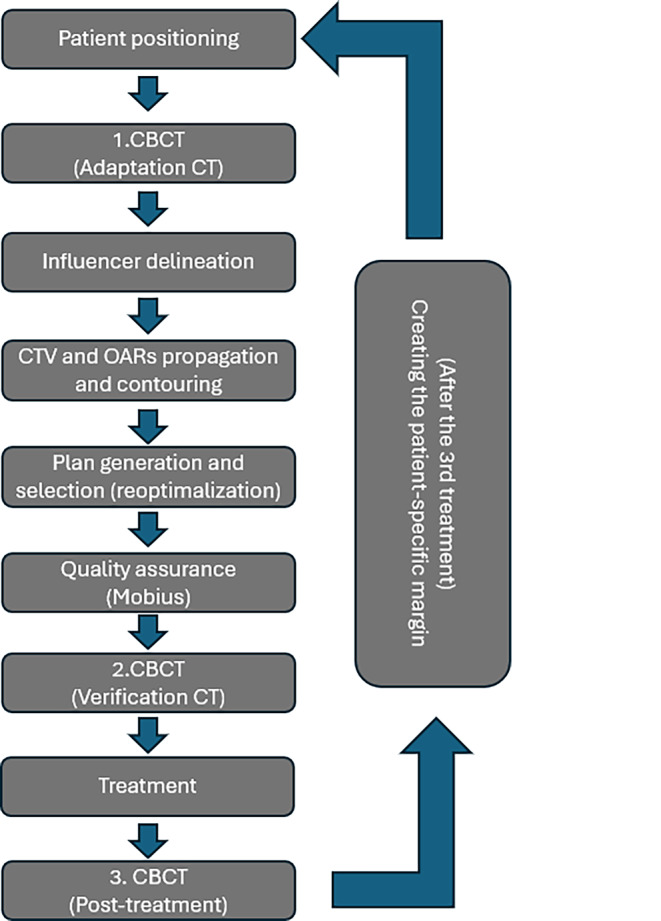
Workflow of the online adaptive re-optimization process, CBCT-cone beam CT, OAR- organs at risk

The planning system then generates two plans. One is based on the planning CT and the original plan but recalculated to the anatomy of the day: the scheduled plan. Another plan is made based on the new contours, considering the preset clinical goals: the adaptive plan. The clinician can decide which plan should be delivered on the day. The plans undergo a quality assurance check using the Mobius 3D System (Varian Medical Systems, Palo Alto, CA, USA). After the adaptation procedure, a second (verification) CBCT is done to check the inter-adaptation changes before the treatment [12]. Additionally, a third (post-treatment) CBCT is acquired for the first three fractions for the calculation of the patient-specific margin. Post-treatment CBCT is prepared also once a week during the whole therapy, immediately following the treatment, which allows us to detect intrafractional changes in the anatomy during the treatment and to verify that the target volume is still covered by the PTV. This online adaptive workflow is summarized in Fig. 1.

As the adaptive treatment planning is based on CBCT and gas artifacts can seriously deteriorate the visibility of organs on CBCT, online adaptive treatment can be complicated or should even be cancelled in some cases. To ensure the safety and effectiveness of adaptive radiotherapy, patients are provided with extensive pretreatment guidance. All treatments are performed with an empty bladder, and patients are required to adhere to strict fluid intake protocols. Specifically, patients should avoid consuming fluids, coffee, or tea starting 1 h prior to the treatment session. For those undergoing concurrent chemotherapy, radiotherapy is administered first to avoid rapid bladder filling and increased urine excretion.

During the treatment course, patients are advised to avoid consuming hard-to-digest or spicy foods. To minimize gas formation, medications such as Simethicone are recommended, beginning at the planning CT stage. If necessary, laxatives are prescribed to maintain optimal image quality throughout the treatment process.

### Retrospective analysis of CBCT images

A total of 239 treatment fractions with 496 CBCT images were collected during the investigation. The CBCT images were imported into the Varian Eclipse treatment planning system (TPS) and analyzed retrospectively. At least two CBCTs were performed for each fraction: an adaptation and a verification CBCT. Any anatomical differences detected during this period are referred to as adaptation changes.

During the study, bladder contouring was performed for each fraction and for each CBCT. According to our protocol for nonadaptive radiation therapy (non-ART) of the bladder, the CTV corresponds to the external contour of the bladder and the macroscopically visible tumor mass plus a 3-mm safety margin, due to the tumor cell spread outside the bladder. For CTV–PTV expansion, an isotropic 1.5-cm margin was determined. The GTV is only contoured when the tumor bed is marked with lipiodol. Following this, the GTV–CTV expansion is 3 mm, and the CTV–PTV expansion for the tumor bed is 8 mm.

In the case of online adaptive radiation therapy (oART), the initial determination CTV is the same as for non-ART treatments. After the initial experiences with oART, it became evident that a new approach was needed for CTV–PTV expansion. Each treatment is based on the contours and plans adjusted to daily anatomy; also, the variations between fractions do not need to be considered. However, considering the increased time required for the adaptation process, a patient-specific CTV–PTV margin focuses on adaptation changes.

**Fig. 2:**
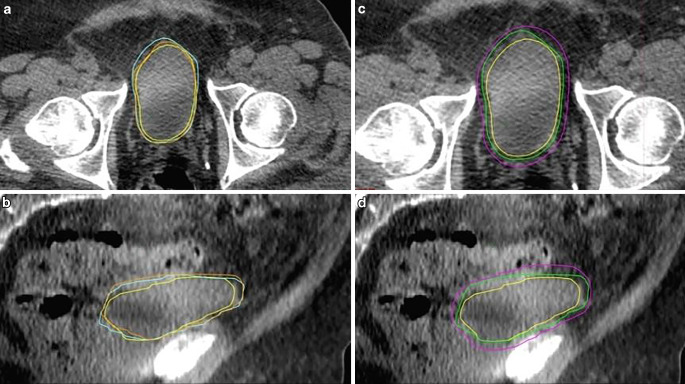
Creating a patient-specific margin. **a**,**b** Bladder contours: *yellow* CBCT 1, *orange* CBCT 2, *blue* CBCT 3. **c**,**d** *Green* union of bladder contours, *purple *contour with patient-specific margin

For the CBCT images of the first three fractions, the contours of the bladder are determined offline. These bladder volumes are merged, and a surrogate PTV is created, expanding the CTV on the first CBCT so that it should cover the union of the bladder volumes. This process is performed for all three fractions, and the largest expansions in all six directions are selected to obtain the patient-specific CTV–PTV margin for the rest of the fractions. Bladder contours drawn on CBCTs 1,2, and 3 with the union bladder contour and creation of the patient-specific PTV margin are shown in Fig. 2.

### Determining volume and coverage differences

The volumes of PTVs created for non-ART and oART were determined per fraction and compared with each other. Two clinically important volumes were determined. The part of the PTV that extends beyond the bladder on the verification CT scan is identified as redundant volume (rV), representing healthy tissue that is unnecessarily irradiated.If the bladder exceeded the PTV in any direction on the verification CT scan, we called it the “missed target volume” (mV). Only volumes above 1 cm^3^ were registered. To assess the accuracy of radiation treatment, we further investigated the relationship between rV and PTV. Differences between treatment types for PTV and rV were analyzed using mixed-effect repeated-measures analysis of variance (ANOVA).

## Results

Due to the conditions of adaptive technology, treatment times were increased compared to conventional radiotherapy. The average duration of treatment from the first CBCT to the verification CBCT, including the contouring of influencer structures and target volumes and producing the new plan, was 14.8 min (range 7–49 min). Patient-specific margins were created and the largest CTV–PTV expansions were applied cranially and anteriorly. The detailed CTV–PTV expansions for the 8 patients in oART are summarized in Table 2.

**Table 2:** Individual margins (in cm) between the clinical target volume (CTV) of the bladder and the planning target volume (PTV) of the bladder created in different directions during online adaptive radiation therapy

Patient	Anterior	Posterior	Caudal	Cranial	Right	Left
1	0.6	0.5	0.5	2.3	1.5	1.6
2	0.7	0.6	0.6	0.8	0.8	0.8
3	1.0	0.9	0.7	1.1	0.9	0.9
4	1.2	1.1	0.5	1.8	1.2	0.7
5	1.7	1.0	0.5	3.0	1.4	1.2
6	0.7	1.0	0.5	1.1	0.4	0.4
7	0.7	0.8	0.5	1.3	0.8	0.7
8	0.9	0.9	0.7	1.1	1.0	0.8
Average	0.9	0.9	0.6	1.6	1	0.9

**Fig. 3:**
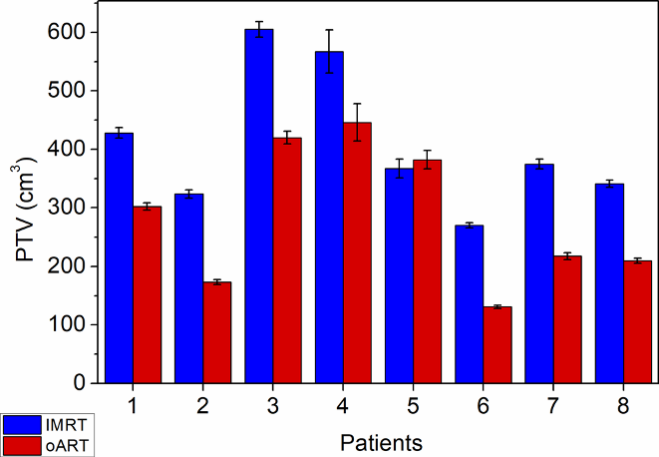
Differences in PTVs by patients for non-ART and oART

The average PTV volume was 416.5 cm^3^ (range 188.2–991.3 cm^3^) for non-ART and 291.8 cm^3^ (range 114.5–810.4 cm^3^) for oART. This represented an overall 29.9% reduction in the PTV. The difference was significant (*p* = 0.0007). Differences in the volumes of the PTVs by patient are summarized in Fig. 3.

**Fig. 4:**
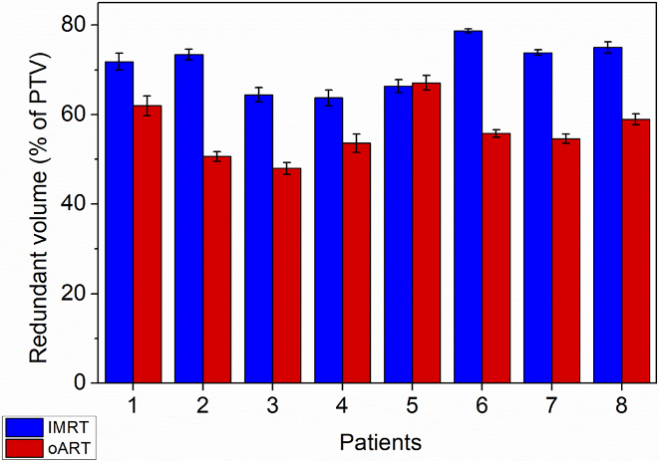
Differences in redundant volumes by patients for IMRT and oART

**Fig. 5:**
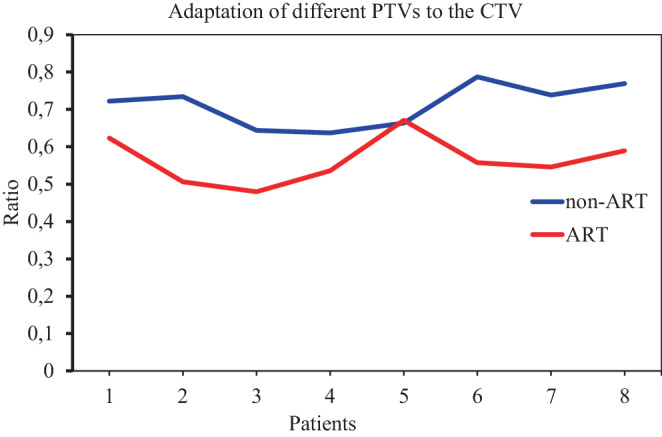
Ratio of rV to PTV for each patient, showing the accuracy of the treatment with non-ART and ART. The lower the ratio, the more conformal the treatment

The average redundant volume (rV) was 285.5 cm^3^ (range 180.2–509.1 cm^3^) for non-ART and 160.1 cm^3^ (range 59.2–338.4 cm^3^) for oART. The difference between the two treatment types was significant (*p* = 0.0011). The volume of irradiated healthy tissue was reduced by 43.9% on average (Fig. 4). The ratio of rV to PTV was 0.71 for non-ART and 0.56 for oART. The lower the ratio, the more accurate the treatment. The relationship between rV and PTV is shown in Fig. 5. With both methods, only a few cases of target volume geometrical miss were observed. With non-ART treatment, there were 12 fractions where the missed volume was more than 1 cm^3^, and with oART, only 4 fractions. The average mV was 11 cm^3^ (range 1.1–56.5 cm^3^) for non-ART and 6.7 cm^3^ (range 1.7–21.2 cm^3^) for oART.

After the first treatments, it became clear that patient cooperation was essential to achieve optimal patient-specific margins. Failure to follow a strict fluid intake protocol during margin creation may result in increased PTV extension. This was the case for patient 5, who did not take the advice of the radiation oncologist and RTT during the first three sessions, and, therefore, a much larger safety margin was created than would have been required for the later sessions. During the treatment, another difficulty was the presence of gas artifacts that severely distorted the image quality during CBCT-based planning. In addition to strict fluid intake constraints, a strict diet and various digestive medications to reduce gas production were used. Despite all these cautionary measures and preparations, in some cases, treatment had to be postponed and delivered on another day.

## Discussion

During pelvic radiotherapy, special attention should be paid to acute and late gastrointestinal side effects, which are determining factors for quality of life [13]. With the advent of the IMRT technique and the reduction of dose to organs at risk, the incidence of acute gastrointestinal side effects could be significantly reduced compared to conformal radiotherapy. Søndergaard et al. found a clear correlation between acute grade 2 (CTCAE 3.0) diarrhea and V45Gy of the small bowel [14]. Currently, there is no consensus regarding dose constraints for the bowels, but a limit of V45Gy < 300 cm^3^ to the small bowel is certainly an important dose–volume parameter related to gastrointestinal toxicity. According to recommendations from Kavanagh et al., to avoid severe acute side effects, it is suggested to keep V45Gy < 195 cm^3^ [14–17]. A comprehensive study examining predictors and constraints of late bowel toxicity after pelvic radiotherapy suggests that increased doses to not only the small bowel but also to the sigmoid colon and large bowel can contribute to the incidence of late bowel toxicity [17]. From these experiences, it can be concluded that reducing radiation exposure to the whole gastrointestinal tract can also reduce acute and late GI side effects.

**Fig. 6:**
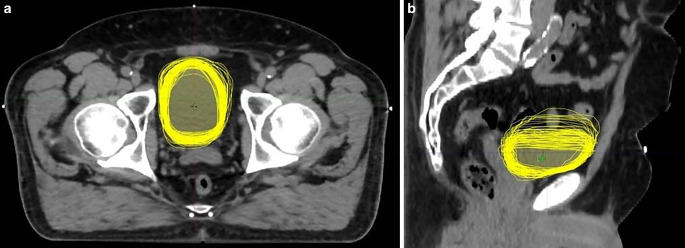
All bladder contours taken during the whole treatment course of a patient, shown on the planning CT. With rigid registration to the bony structures. **a** Axial view, **b** sagittal view

However, due to the variability in bladder shape and volume, IMRT with image-guided radiotherapy for bladder cancer requires a large safety margin. Most commonly, an isotropic extension of 1.5–2 cm is used, as in the BC2001 study [18]. Previous studies have found that an isotropic safety margin of 1.5–2 cm is inadequate, and in some cases, anisotropic margins are needed—much larger in caudal and anterior directions than posteriorly and inferiorly [16]. This was also observed during our treatments. All bladder contours during the whole treatment course for one patient projected onto the treatment planning CT are shown in Fig. 6. The created individual margins clearly show that larger extensions were used in the cranial direction, while caudally, one third of the conventional extension was sufficient.

Previous studies have shown that adaptive techniques can safely reduce the PTV. Several studies have demonstrated the effectiveness of PoD and composite volume, but there is limited information available about ReOpt [9].

Kong et al. concluded that all adaptive strategies significantly reduced the irradiated volume compared to the standard treatment. Re-optimalization was the most efficacious among the strategies. The median reduction of the PTV (the volume irradiated with an accumulated dose of 43.7 Gy [95% of prescription dose]) was 29% and 12%, in reOpt and PoD, respectively [20]. Vestergraad et al. found similar results [18]. ReOpt being the superior technique compared to nonadaptive RT, the volume receiving more than 57 Gy (corresponding to 95% of the prescribed dose) was reduced to 66% (range 48–100%) for plan selection and to 41% (range 33–50%) for ReOpt [21]. In these two studies, a margin of only 5 mm between the CTV and the PTV was used. This could lead to a greater reduction in the PTV.

We know of three studies in which ReOpt adaptive radiotherapy was performed with a patient-specific margin. In two of these, the patient-specific margin was determined using CBCT data from the first 3–5 fractions. In one, Silbot et al. achieved a 47% reduction in the PTV [22], while Aström et al. reduced V95 by 33.9% [23]. In a recent study, a simultaneously integrated boost technique was implemented with high accuracy in daily online adaptive radiotherapy using fiducial markers. The authors used a 5-mm CTV–PTV expansion in the first treatments. If the bladder on the second CT was completely encompassed by this expansion, a margin of 7 mm was used. In any direction in which the uniform 5‑mm expansion did not encompass the bladder, the necessary expansion to encompass the bladder was determined. With this technique, the bladder was inside the PTV in 90% of all fractions for all patients [24].

**Fig. 7:**
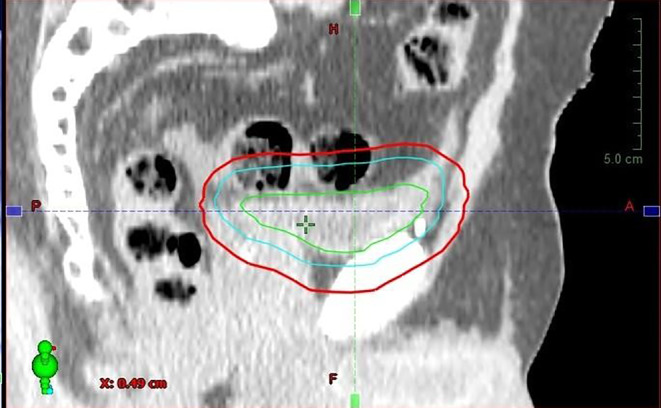
PTV reduction for one patient. *Green *bladder contour (CBCT 1), *blue *oART PTV, *red* non-ART PTV

In the current research, based on the experiences with initial treatments, we conclude that the 5‑mm safety margin is insufficient due to the time-consuming adaptive workflow. We have developed patient-specific margins that consider intrafractional changes. This technique reduced the PTV by an average of 29.9% in the studied population, which is clearly shown in Fig. 7. Due to the individualized anisotropic expansion, we managed to reduce the irradiated volume of healthy tissues by 43.9%. In addition to the reduction in PTV volume, the individual margin also reduced the incidence of target volume misses, thus providing a more accurate treatment overall. This is almost in parallel to the results of previous studies.

The CBCT-guided oART demonstrated significant sparing of OARs, such as the small bowel and rectum. For example, one study reported a reduction in the volume of normal tissue receiving more than 45 Gy by 29% with adaptive techniques compared to conventional methods. This reduction in dose exposure translates into lower risks of acute and late toxicities for patients. Follow-up assessments showed that patients treated with oART experienced minimal acute side effects, with some common issues being manageable symptoms like increased urination and mild urinary tract discomfort. Importantly, no severe late toxicity was reported over a 2-year follow-up period, thus reinforcing the safety profile of this adaptive radiotherapy approach [25].

## Conclusion

Based on the studied patient population, we have demonstrated that with our local protocol, online adaptive re-optimizing bladder irradiation is safe and more accurate than conventional therapy. Adaptation requires high-level patient education and cooperation (strict constraints on fluid intake and dietary guidelines). Due to the increased treatment time and requirement for assistance from highly trained personnel, oART is suitable for the daily care of only a small number of patients. The clear toxicity advantage of oART needs to be investigated in prospective studies.

## Data Availability

All data of the study are stored on a secure network, and the corresponding author can authorize access to view them.

## References

[1] Kiss B, Burkhard FC, Thalmann GN (2016) Open radical cystectomy: still the gold standard for muscle invasive bladder cancer. World J Urol 34(1):33–39 (Jan 1) 26586476 10.1007/s00345-015-1729-7

[2] Tree AC, Jones K, Hafeez S, Sharabiani MTA, Harrington KJ, Lalondrelle S et al (2018) Dose-limiting Urinary Toxicity With Pembrolizumab Combined With Weekly Hypofractionated Radiation Therapy in Bladder Cancer. Int J Radiat Oncol Biol Phys 101(5):1168–1171 30012528 10.1016/j.ijrobp.2018.04.070

[3] Marcq G, Souhami L, Cury FL, Salimi A, Aprikian A, Tanguay S et al (2021) Phase 1 Trial of Atezolizumab Plus Trimodal Therapy in Patients With Localized Muscle-Invasive Bladder Cancer. Int J Radiat Oncol Biol Phys 110(3):738–741 33421558 10.1016/j.ijrobp.2020.12.033

[4] Fokdal L, Honoré H, Høyer M, Meldgaard P, Fode K, Von Der Maase H (2004) Impact of changes in bladder and rectal filling volume on organ motion and dose distribution of the bladder in radiotherapy for urinary bladder cancer. Int J Radiat Oncol Biol Phys 59(2):436–444 (Jun 1) 15145160 10.1016/j.ijrobp.2003.10.039

[5] Dees-Ribbers HM, Betgen A, Pos FJ, Witteveen T, Remeijer P, Van Herk M (2014) Inter- and intra-fractional bladder motion during radiotherapy for bladder cancer: A comparison of full and empty bladders. Radiother Oncol 113(2):254–259 (Nov 1) 25483834 10.1016/j.radonc.2014.08.019

[6] Foroudi F, Wong J, Haworth A, Baille A, McAlpine J, Rolfo A, Kron T, Roxby P, Paneghel A, Williams S, Duchesne G, Tai K (2009) Offline adaptive radiotherapy for bladder cancer using cone beam computed tomography. J Med Imag Rad Onc 53(2):226–233 10.1111/j.1754-9485.2009.02066.x 19527372

[7] Hijab A, Tocco B, Hanson I, Meijer H, Nyborg CJ, Bertelsen AS, Smeenk RJ, Smith G, Michalski J, Baumann BC, Hafeez S (2021) MR-Guided Adaptive Radiotherapy for Bladder Cancer. Front Oncol 11:637591 33718230 10.3389/fonc.2021.637591 PMC7947660

[8] Kibrom AZ, Knight KA (2015) Adaptive radiation therapy for bladder cancer: a review of adaptive techniques used in clinical practice. J of Medical Radiation Sci 62(4):277 10.1002/jmrs.129 PMC4968556 27512574

[9] Kong V, Hansen VN, Hafeez S (2021) Image-guided Adaptive Radiotherapy for Bladder Cancer. Clin Oncol 33(6):350–368 (Jun 1) 10.1016/j.clon.2021.03.023 33972024

[10] Choudhury A, Porta N, Hall E, Song YP, Owen R, MacKay R et al (2021) Hypofractionated radiotherapy in locally advanced bladder cancer: an individual patient data meta-analysis of the BC2001 and BCON trials. Lancet Oncol 22(2):246–255 (Feb 1;) 33539743 10.1016/S1470-2045(20)30607-0 PMC7851111

[11] Wegener S, Schindhelm R, Tamihardja J, Sauer OA, Razinskas G (2023) Evaluation of the Ethos synthetic computed tomography for bolus-covered surfaces. Phys Medica 113(1):102662 (Sep) 10.1016/j.ejmp.2023.102662 37572393

[12] Hu Y, Byrne M, Archibald-Heeren B, Collett N, Liu G, Aland T (2020) Validation of the preconfigured Varian Ethos Acuros XB Beam Model for treatment planning dose calculations: A dosimetric study. J Appl Clin Med Phys 21(12):27–42 (Dec 1) 33068070 10.1002/acm2.13056 PMC7769396

[13] Feuerstein MA, Goenka A (2015) Quality of Life Outcomes for Bladder Cancer Patients Undergoing Bladder Preservation with Radiotherapy. Curr Urol Rep 16(11):1–6 (Nov 10) 26343030 10.1007/s11934-015-0547-1

[14] Sondergaard J, Holmberg M, Jakobsen AR, Agerbæk M, Muren LP, Hoyer M (2014) A comparison of morbidity following conformal versus intensity-modulated radiotherapy for urinary bladder cancer. Acta Oncol 53:1321–1328 24980045 10.3109/0284186X.2014.928418

[15] Kavanagh BD, Pan CC, Dawson LA, Das SK, Li XA, Ten Haken RK et al (2010) Radiation Dose-Volume Effects in the Stomach and Small Bowel. Int J Radiat Oncol 76(3):S101–7 (Mar 1) 10.1016/j.ijrobp.2009.05.071 20171503

[16] Mcdonald F, Waters R, Gulliford S, Hall E, James N, Huddart RA (2015) Defining bowel dose volume constraints for bladder radiotherapy treatment planning. Clin Oncol: 22–29 (Jan) 10.1016/j.clon.2014.09.016 25445550

[17] Jadon R, Higgins E, Hanna L, Evans M, Coles B, Staffurth J (2024) A systematic review of dose-volume predictors and constraints for late bowel toxicity following pelvic radiotherapy. Radiat Oncol 14(1):1–14 (2019 Apr 3) 10.1186/s13014-019-1262-8 PMC6448293 30943992

[18] Huddart RA, Hall E, Hussain SA, Jenkins P, Rawlings C, Tremlett J et al (2013) Randomized noninferiority trial of reduced high-dose volume versus standard volume radiation therapy for muscle-invasive bladder cancer: Results of the BC2001 Trial (CRUK/01/004). Int J Radiat Oncol Biol Phys 87(2):261–269 (Oct) 23958147 10.1016/j.ijrobp.2013.06.2044 PMC3753507

[19] Fonteyne V, Sargos P (2021) What is the Optimal Dose, Fractionation and Volume for Bladder Radiotherapy? Clin Oncol 33(6):e245–50 (Jun 1) 10.1016/j.clon.2021.03.013 33832838

[20] Kong VC, Taylor A, Chung P, Craig T, Rosewall T (2019) Comparison of 3 image-guided adaptive strategies for bladder locoregional radiotherapy. Med Dosim 44(2):111–116 (Jun 1) 29655582 10.1016/j.meddos.2018.03.004

[21] Vestergaard A, Muren LP, Søndergaard J, Elstrøm UV, Høyer M, Petersen JB (2013) Adaptive plan selection vs. re-optimisation in radiotherapy for bladder cancer: a dose accumulation comparison. Radiother Oncol 109(3):457–462 (Dec) 24100147 10.1016/j.radonc.2013.08.045

[22] Sibolt P, Andersson L, Calmels L, Sjostrom D, Behrens CF, Lindberg H et al (2020) Results of a Pilot Study on Online Adaptive Radiotherapy of Bladder Cancer with Artificial Intelligence-driven Full Re-optimization on the Anatomy of the Day. Int J Radiat Oncol 108(3):79–80 (Nov 1)

[23] Åström LM, Behrens CP, Calmels L, Sjöström D, Geertsen P, Mouritsen LS et al (2022) Online adaptive radiotherapy of urinary bladder cancer with full re-optimization to the anatomy of the day: Initial experience and dosimetric benefits: oART of bladder cancer: initial experience and dosimetric benefits. Radiother Oncol 171:37–42 (Jun 1) 35358605 10.1016/j.radonc.2022.03.014

[24] Azzarouali S, Goudschaal K, Visser J, Hulshof M, Admiraal M, van Wieringen N et al (2023) Online adaptive radiotherapy for bladder cancer using a simultaneous integrated boost and fiducial markers. Radiat Oncol 18(1) (Dec 1) 10.1186/s13014-023-02348-8 PMC10557331 37803392

[25] Azzarouali S, Goudschaal K, Visser J, Bel A, Daniëls L, den Boer D (2024) Cone-Beam Computed Tomography-Guided Online Adaptive Radiotherapy: Promising Results for Bladder Cancer Case. Cureus 16(9):e68863. 10.7759/cureus.68863 (Sep 7) 39376847 10.7759/cureus.68863 PMC11457903

